# A Polyimide Film-Based Simple Force Plate for Measuring the Body Mass of Tiny Insects

**DOI:** 10.3390/s22218352

**Published:** 2022-10-31

**Authors:** Kenichiro Shimazaki, Takumi Sugimoto, Hirofumi Toda, Hidetoshi Takahashi

**Affiliations:** 1 Department of Mechanical Engineering, Faculty of Science and Technology, Keio University, Yokohama 223-8522, Japan; 2 International Institute for Integrative Sleep Medicine, University of Tsukuba, Ibaraki 305-8575, Japan

**Keywords:** force plate, fruit fly, body mass measurement, laser processing

## Abstract

Insects exhibit excellent maneuvers such as running and flying despite their small bodies; therefore, their locomotion mechanism is expected to provide a design guideline for micromachines. Numerical simulations have been performed to elucidate this mechanism, whereby it is important to develop a model that is physically identical to the target insect’s parts to reproduce kinematic dynamics. In particular, in flight, the shape and mass of wings, which flap at high frequencies, are significant parameters. However, small insects such as fruit flies have small, thin, and light wings; thus, their mass cannot be easily measured. In this study, we proposed a high-resolution and simple force plate to measure the mass of each part of a tiny insect. The device consists of a circular plate supported by flat spiral springs made of polyimide film, and a laser displacement meter that detects the displacement of the center of the plate. The simple plate fabrication process requires only a couple of minutes. A fabricated force plate with a sub-N/m spring constant achieved a resolution of less than 2 µg. As a demonstration, the wing mass of the fruit flies was measured. The experimental results suggest that the wings accounted for approximately 0.4% of the body mass.

## 1. Introduction

Many micromachines with various locomotion capabilities such as walking, swimming, and flying have been developed [1,2,3,4,5]. These small machines require an optimum design and force control according to their size because of the scale effect. In nature, insects are capable of high maneuverability and stability for their respective locomotion, despite their small body sizes. Thus, their kinematic dynamics are expected to provide design guidelines for micromachines.

Several studies have been conducted to understand the mechanisms of insect locomotion. Among them, microelectromechanical systems (MEMS) are force sensors which have been used to measure the specific force of tiny insects, such as the ground reaction forces of fruit flies, ants, and cockroaches [6,7,8], and flight forces of fruit flies [9,10,11]. These devices are sufficiently small to measure force precisely without affecting the insects’ original locomotion. In addition, numerical simulations have also been conducted, particularly for insect flights [12,13,14,15,16,17]. In the simulations, any aerodynamics related to insect locomotion can easily be calculated without complicated setups or sensors. Therefore, they have helped us to systematically understand insect flight dynamics. It is important to develop a model that is physically identical to the target insect parts to reproduce the actual kinematic dynamics. In particular, insect wings are thin and have a low mass, making it difficult to measure their mass and other mechanical characteristics.

The conventional method for measuring the mass of insects uses an electronic balance. However, a high-resolution balance is expensive and is delicate to the impulsion. In the case of conventional MEMS force sensors such as those described above, the sensor can be designed to achieve sufficiently high sensitivity. However, it is more fragile and laborious to fabricate. Thus, it is desirable to develop a force plate that can accurately measure the mass of small insect parts with a simple production process, high durability, and low price.

Herein, we propose a force plate suitable for measuring the mass of a fruit fly with a body mass of approximately 1 mg. The proposed device consists of a circular plate structure with supporting spiral springs made of polyimide film, which is a stable material with excellent processability, and a laser displacement meter. The circular plate is displaced vertically via the force applied to the plate, and the displacement is detected by a laser displacement meter. Subsequently, the applied force is calculated from the displacement. In the case of the proposed structure, the local strain of any part under an applied force was rather small, enabling the force plate to be both tough and highly sensitive. Additionally, because the plate and laser displacement meter do not contact each other, the unexpected collisional force on the force plate does not adversely damage the laser displacement meter.

In this study, a polyimide film-based force plate was designed and fabricated. The fabrication process consisted of cutting the polyimide film using a UV laser cutter. The mechanical properties of the force plates were evaluated. After calibration, we measured the mass of the body, head, thorax, and wings of 16 fruit flies.

## 2. Design, Principle, and Development

### 2.1. Design and Principle

To accurately measure the mass of the fruit flies, including the wing mass, the resolution of the force plate should be less than 1/100 of the total mass, which is approximately 0.01 mg. Therefore, the spring constant should be small enough to achieve the required resolution. The plate size must also be sufficiently large, such that a fruit fly can be easily placed on it. However, as the spring constant of the force plate decreases, the noise caused by the vibration or airflow also increases. Similarly, as the area of the plate and springs increases, the noise caused by the airflow becomes relatively large as the larger plate area captures more airflow. In addition, the resonant frequency decreases as the plates are large and the spring constant is small, resulting in low responsiveness. Considering these tradeoffs, the force plate dimension was chosen as 30 mm to enable convenient placement of the flies, with a spring constant of the order of sub-N/m.

**Figure 1:**
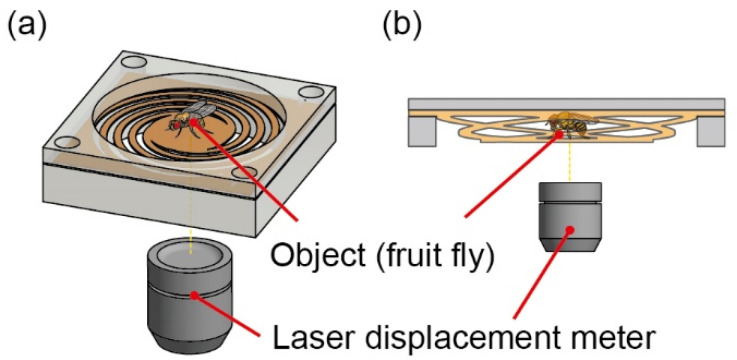
Schematics of the device’s in (**a**) bird’s-eye view and (**b**) side view.

The proposed device is illustrated in Figure 1. The device consists of a force plate made of polyimide film and a laser displacement meter. Apart from being a cheap, easily available, and stable material, polyimide film has excellent processability. Placing an object on the central plate caused the four spiral springs supporting the plate to deform vertically, and the displacement was measured using a laser displacement meter fixed under the force plate. Spiral springs that produce large displacements can be densely arranged using a spiral shape for the four springs that support the central plate. Additionally, the spiral shape is easier to change parameters. The mass of the object can be measured by multiplying the premeasured spring constant with the displacement. The workability and viscoelasticity of polyimide make the force plate durable and easy to fabricate.

**Figure 2:**
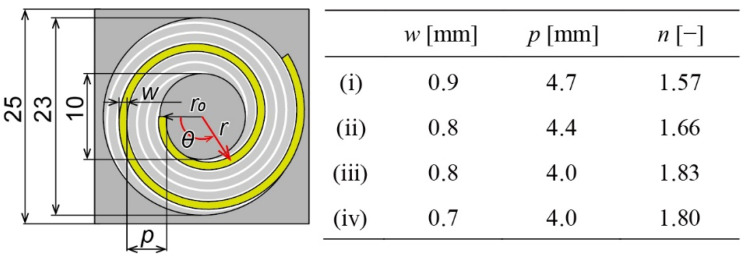
Detailed design and parameters of four types of force plate.

The design details of the spiral-coil spring are shown in Figure 2. The overall dimensions were 25 × 25 mm, and the central plate was circular with a diameter of 10 mm. The spiral coil had the same shape as the Archimedes spiral. The four coils were equally spaced with a phase difference of 90° and did not interfere with each other. The longer the length of the spiral, the smaller the spring constant.

We define the radius at any point on the spiral as *r* [mm] and the central angle as *θ* [rad]. When coil width *w* [mm] and pitch *p* [mm] are determined, the relationship between *r* and *θ* is given by Equations (1) and (2).

(1) $$r=5-\frac{w}{2}+\frac{p\;\theta }{2\;\pi }\;\left(0\leq \theta \leq \theta_{max}\right)$$

(2) $$\theta_{\max}=\frac{6.5+w}{p}\times 2\pi$$

The number of turns in the spiral, denoted by *n* is given by Equation (3).

(3) $$N=\frac{\theta_{\max}}{2\pi }$$

**Figure 3:**
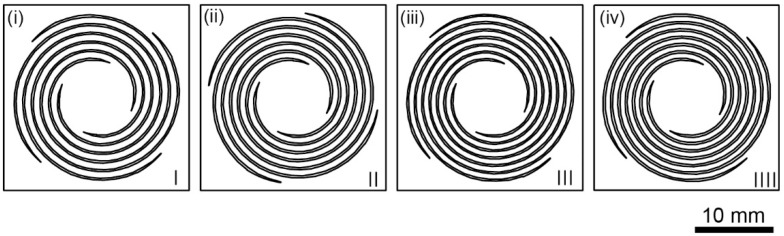
Design drawings of each force plate according to the parameters shown in Figure 2. The coil width *w*, pitch *p*, and turn *n* differ from each other. The order of (i), (ii), (iii), and (iv) are corresponding to those of Figure 2.

We decided on four different types (i)–(iv), as shown in Figure 2, and each design drawing is shown in Figure 3. As the type number increases, the spiral becomes thinner and longer, thereby reducing the spring constant.

The spring constants of each design and the resonant frequencies were evaluated using a finite element method (FEM) simulation (COMSOL Multiphysics 5.6, COMSOL). We designed the force plate structure by trial and error using an FEM simulation to achieve the required specifications. We used a polyimide film with a thickness of 100 µm, Young’s modulus of 3.4 GPa, and density of 1420 kg/m^3^. In the simulation, the outer region (area bounded by a 25 mm square and 23 mm circle) was fixed and a force was applied to the entire plate surface equally. The spring constant is calculated by dividing the maximum displacement by the input force. The calculated values for each characteristic are listed in Table 1.

**Figure 4:**
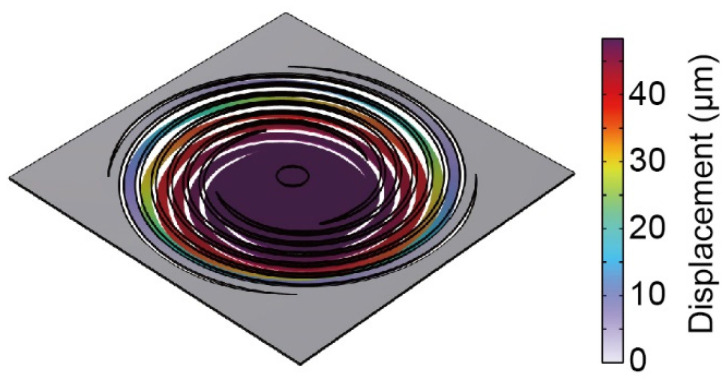
Displacement distribution by simulation of the force plate (iv).

Figure 4 shows the displacement distribution of type (iv). In the simulation, a force of 10 µN was applied uniformly across the plate and the spring constant was calculated from the maximum displacement. The simulation results showed that the spring constants ranged from 0.20 to 0.32 N/m, which satisfies the required specification. The resonant frequency was calculated to be 13.6 to 16.6 Hz.

**Figure 5:**
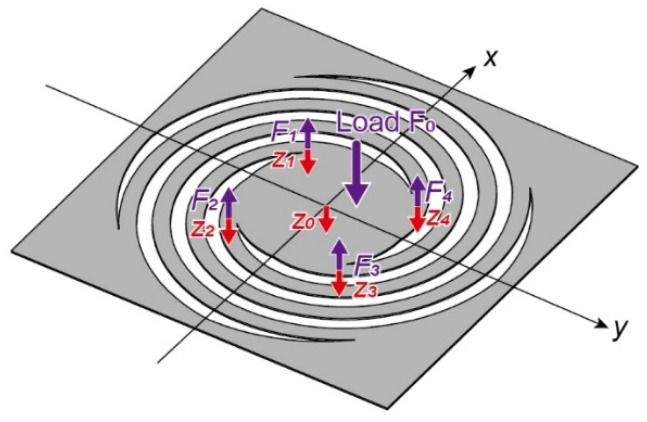
Relationship between the applied force and output displacement.

For cantilever beams, the magnitude of deformation is proportional to the magnitude of the load applied to the end of the beam [18]. The designed spring was also expected to respond linearly to the load magnitude. We define each spiral of the spring coil as a cantilever beam with spring constant *k’*. The total force applied to the plate and the plate center displacement were defined as *F*_0_ and *z*_0_, respectively. Additionally, the force applied to the edge of each spiral was *F*_1_–*F*_4_, and the vertical displacement of the edge of each spiral was *z*_1_–*z*_4_, as shown in Figure 5.

From the force-balancing relationship and Hooke’s law, the following equation is valid:

(4) $$F_{0}=F_{1}+F_{2}+F_{3}+F_{4}=k\prime \left(z_{1}+z_{2}+z_{3}+z_{4}\right)$$

Providing that the central plate is rigid, the following equation is also valid:

(5) $$z_{0}=\frac{z_{1}+z_{3}}{2}=\frac{z_{2}+z_{4}}{2}$$

From Equations (4) and (5), we can calculate the following:

(6) $$F_{0}=4k^{\prime }z_{0}$$

From Equation (6), when the central plate is rigid, the total force applied to the plate can be calculated from the center displacement and the spring constant alone [19].

### 2.2. Fabrication

**Figure 6:**
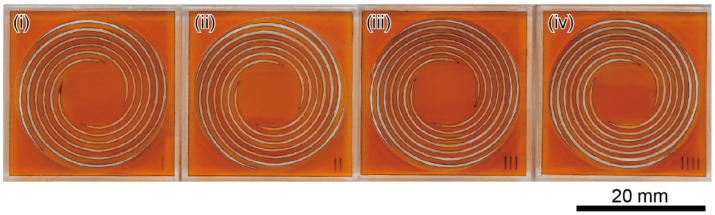
Photograph of the fabricated polyimide force plates according to the parameters and design of (i)–(iv) given in Figure 2 and Figure 3.

Four types of force plates were fabricated using a UV laser microprocessing machine (OLMUV-355-5A-K; OPI, Calabasas, CA, USA). A 100-µm-thick polyimide film was used as the starting material. Force plates were fabricated within seconds to tens of seconds per plate. When the cutting was inadequate, they were manually separated using tweezers. Photographs of the fabricated force plates are shown in Figure 6.

**Figure 7:**
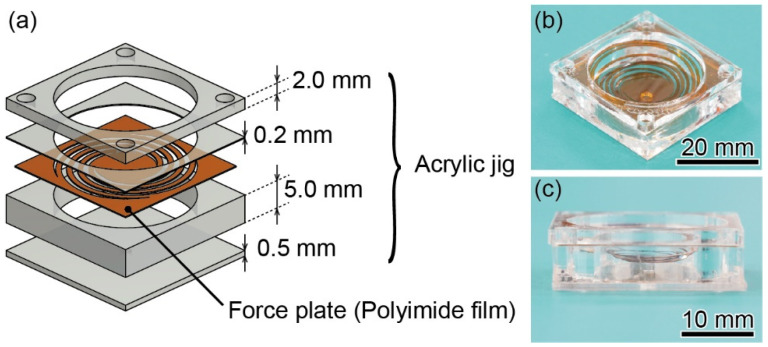
(**a**) Diagram of the assembly of the device (**b**,**c**) photographs of the assembled device.

As shown in Figure 7a, the force plate was fixed on a 5-mm-thick acrylic jig with a hole drilled in the center using a UV-cured adhesive (Optical Adhesive 61, Norland Products). A 2.0-mm-thick acrylic jig of the same shape was glued on top of the force plate. To block airflow, a 0.5-mm-thick acrylic jig with a 2-mm hole in the center was glued to the bottom. Figure 7b,c shows photographs of the assembled device. The device dimensions were 27 mm^2^ × 7.8 mm, whereas the polyimide force plate was 25 mm^2^.

## 3. Experiment and Results

### 3.1. Calibration

**Figure 8:**
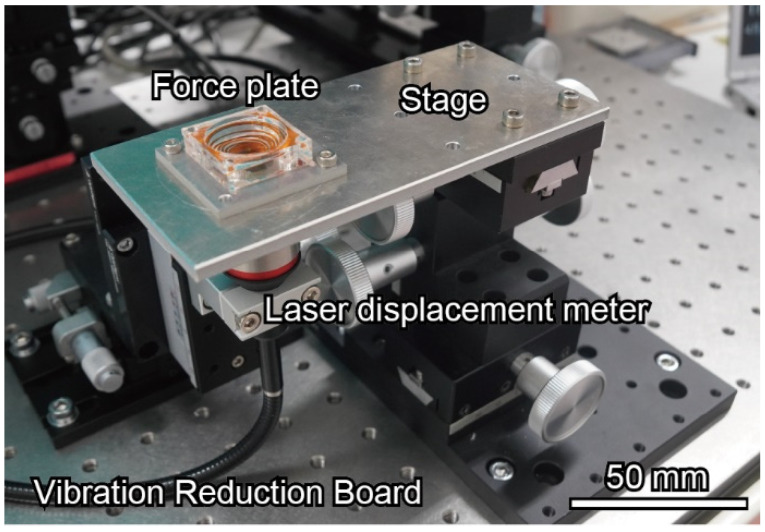
Setup for the calibration and experiment.

The experimental setup for the calibration is shown in Figure 8. The fabricated force plate was fixed on a movable stage and adjusted to align the laser beam with the center of the plate. First, the spring constant was measured by placing calibration weights (1, 2, 5, and 10 mg) on each force plate. In the experiment, the displacement of the plate center was measured using a laser displacement meter (CL-L015 and CL-3050, Keyence Corporation, Osaka, Japan) with a resolution of less than 10 nm. The diameter of the spot was 300 μm, which was smaller than that of the plate structure. The output of the laser displacement meter was processed using a low-pass filter of 1 Hz.

**Figure 9:**
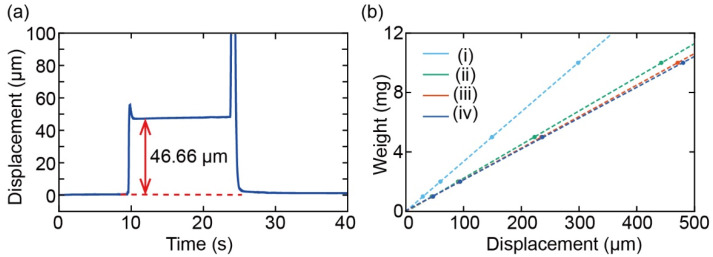
(**a**) Displacement output of the calibration and (**b**) relationship between weight and displacement.

Figure 9a shows the output of the laser displacement meter when a 1 mg weight was placed on plate type (iv). This was used for the demonstration after calibration. The relationship between the mass of the applied weight and the displacement is shown in Figure 9b, where it can be seen that the displacement is proportional to the weight. The spring constants were calculated to be 0.329, 0.221, 0.208, and 0.205 N/m, respectively, from the gradient of the line of best fit.

**Figure 10:**
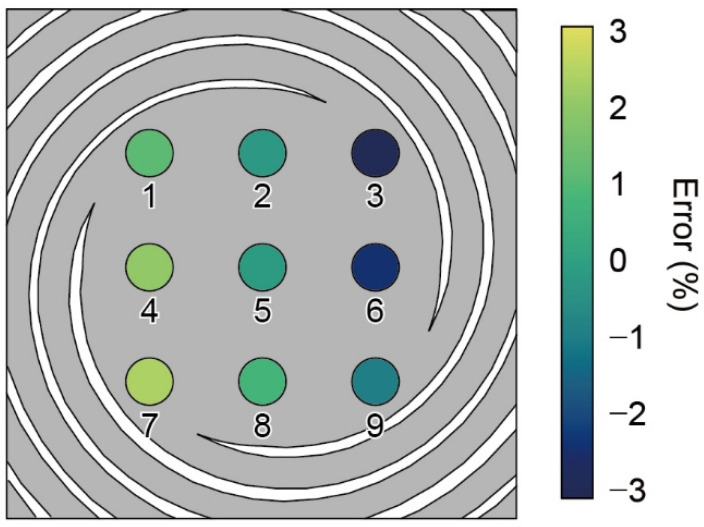
Distribution of the spring constant error of type (iv) in the experiment.

Theoretically, the displacement of the plate center remains the same regardless of where the force is applied. In practice, displacement may vary depending on the location of the applied force [19]. We experimentally evaluated the distribution of the spring-constant error of the force plate. For the experiment, we divided the plate into nine locations (places 1–9) and placed a 1 mg weight on each location. The type (iv) force plate was evaluated, as shown in Figure 10. In the experiment, we defined the average value of nine locations as the reference value. Provided that the error value was positive, the displacement was larger than the average value, and the spring constant was inversely smaller. The displacement varied by approximately ±3%, which was sufficiently low for the measurements. The error distribution suggested that places 3 and 7 were the most insensitive and sensitive, respectively. In addition, the smallest error was found at the center (place 5).

This tendency was thought to be because the spring constant of each spiral was slightly different owing to machining errors or because the laser displacement meter was slightly shifted from the plate center. The experimental results suggest that the spiral spring starting from place 7 had the softest structure, or that the location of the laser displacement meter was closer to place 7.

**Figure 11:**
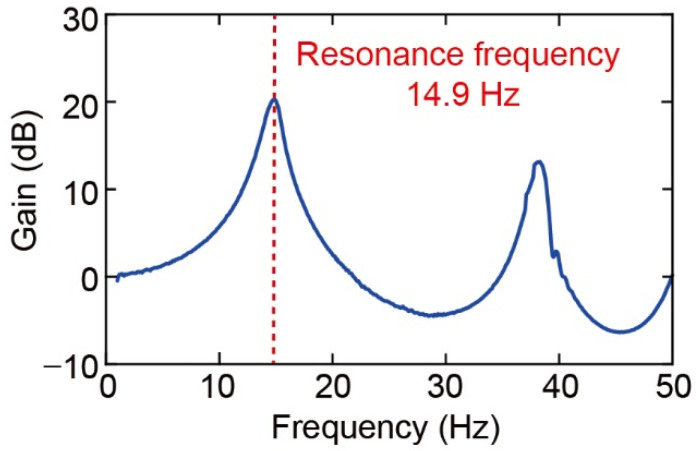
Frequency response of the force plate.

Subsequently, the resonant frequency was evaluated as a mechanical property via an optical heterodyne (NEOARK, MLD-221 D-DWT) and a frequency response analyzer (FRA) (NF, FRA5087). The force plate was attached to a shaker that vibrated from 0.1 to 50 Hz in 0.1 Hz increments (Mini-shaker Type 4810, Brüel and Kjær). Figure 11 shows the frequency response of the type (iv) force plate. The graph shows the amplitude of the plate center with reference to the outside of the force plate. The experiment result showed that the first resonant frequency was 14.9 Hz.

The experimental spring constants and resonant frequencies of the four force plate types are listed in Table 2. The experimental results were similar to the simulated values as shown in Table 1. From all calibrations, the fabricated structures have a sub-N/m spring constant and the resonant frequency exceeds 14.3 Hz, which seems to be adequate for measuring the mass of fruit flies, which have body masses of approximately 1 mg.

### 3.2. Measurement of Body Parts of Fruit Flies

**Figure 12:**
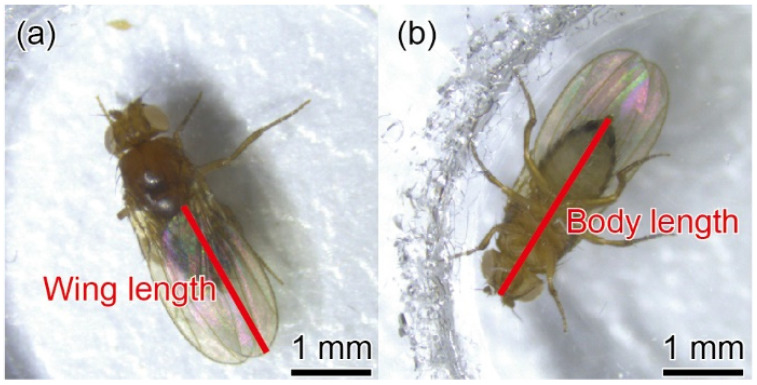
Photographs of (**a**) backside and (**b**) abdominal side of a fruit fly.

To demonstrate the usefulness of the fabricated device, we measured the body mass of fruit flies and the mass of each body part using the fabricated force plate (iv), which is the most sensitive of all the design types. Before using the device, we measured the noise level by calculating the root mean square (RMS) displacement of the empty plate. The noise level was evaluated as less than 0.1 μm, which was equivalent to 2 μg. This value was defined as the force resolution of the device. First, we obtained photographs of each fruit fly to determine whether it was male or female. The body and wing lengths were also measured. Subsequently, the body mass of a single fruit fly was measured. The fruit fly is anesthetized with CO_2_ gas and moved onto a force plate with tweezers while sleeping. After removing the fly from the plate, it was reweighed again by the same process. After that, the wings were removed from the body and the total masses of the right and left wings were measured by placing them softly on the plate. Finally, the head was dissected from the body and the mass of each part was measured by placing the head and abdomen on the plate. Figure 12 shows photographs of the fruit fly used in the experiment and the definition of body and wing length.

**Figure 13:**
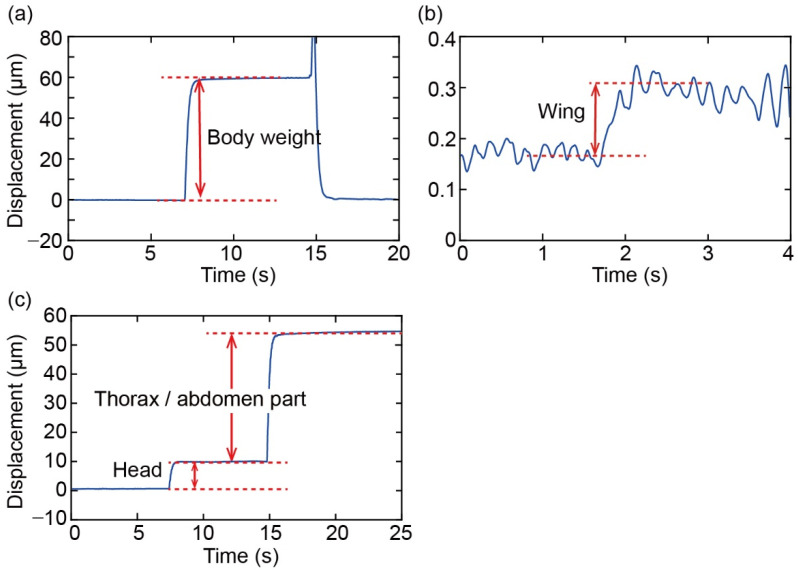
Displacement output of measuring (**a**) body mass, (**b**) wing mass, and (**c**) head and thorax/abdomen mass of a fruit fly.

Figure 13a,b shows the responses of body mass and wing mass measurements, respectively, when each target was placed on the plate. Body mass was measured more than twice and the average was calculated. The average variation in measurements between the two weight measurements for each fruit fly was 0.93%. Wing mass was measured by placing both the right and left wings simultaneously on the plate. The graph suggests that the measured body mass and wing mass were 1.25 mg and 0.0029 mg, respectively. Figure 13c also shows the response of the head and thorax/abdominal mass measurements. The measured head mass and thorax/abdomen mass became 0.19 mg and 0.93 mg, respectively. For disassembling, we used dissecting tweezers and a stereo microscope. Each part was then placed on a force plate as soon as it was disassembled. The experiment was performed on 16 fruit flies (8 males and 8 females).

**Figure 14:**
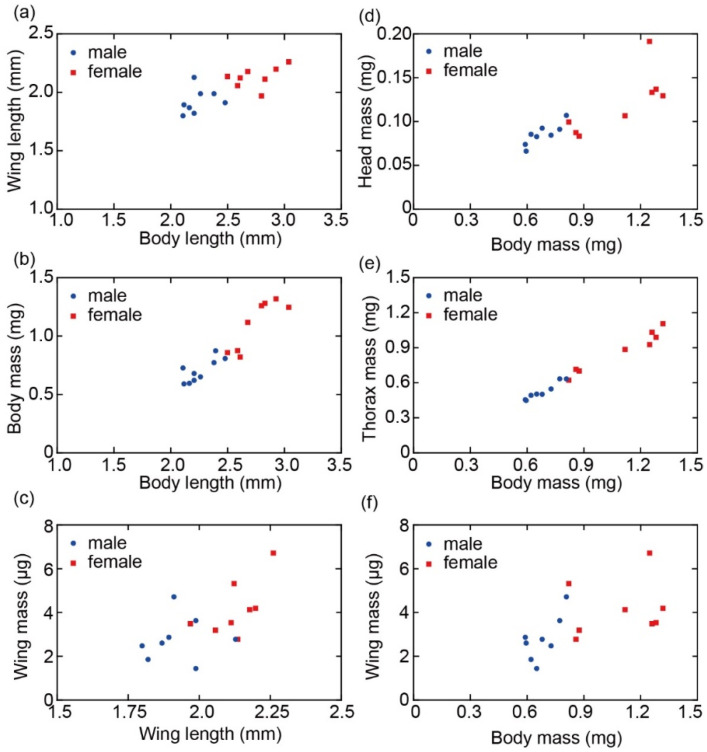
Relationship between (**a**) body length and wing length, (**b**) body length and body mass, (**c**) wing length and wing mass, (**d**) body mass and head mass, (**e**) body mass and thorax/abdomen mass, and (**f**) body mass and wing mass.

From the photographs, the relationship between the body length and wing length was clarified. Figure 14a shows that the body length ranged from 2.1 to 3.0 mm and the wing length ranged from 1.8 to 2.3 mm with a correlation number of 0.76. Figure 14b shows the relationship between the body length and body mass. Body length increased as body mass increased. Similarly, Figure 14c shows the relationship between wing length and wing mass, indicating that the longer the wing length, the heavier the wing mass, although the correlation was not as strong. The distribution of male and female plots suggests that females were larger and heavier than males.

Figure 14d–f shows the relationship between body mass and the head, thorax/abdomen, and wing mass. The correlation between head, thorax/abdomen mass, and body mass was approximately proportional to each other, although the correlation was weaker for the wings and body mass due to the large measurement error.

The average values of male and female measurements are summarized in Table 3. The average body mass of males and females was 0.702 mg and 1.09 mg, respectively. From these experimental results, it was observed that females were larger than males in body length, and weighed approximately 1.55 times more than males.

Comparing the mass of each part, females had a larger proportion of thorax/abdominal mass than males, with the thorax weighing approximately 7.2 times as much as the head in females, while weighing 6.2 times as much in males. Wing mass accounted for approximately 0.4% of body mass in both males and females. The total mass of each part was approximately 90% of the body mass and the remaining 10% was considered to have been lost during disassembly.

## 4. Discussion

High-resolution 3D printers are also applicable in the fabrication of force plate structures based on the aforedescribed principle. However, there is a limit to realizing a small spring constant so that the force plate deforms under its own weight, similar to our developed device. Compared to the spring constant of previously 3D-printed highly sensitive force plate with a spring constant of 5.98 N/m [19], that of our developed device was approximately 30 times lower.

From the experimental results, we demonstrated that the proposed device enabled accurate body part mass measurement of a single fruit fly. The experimentally obtained mass values are expected to be useful as parameters in simulations of the flapping flight of fruit flies. For example, wing mass is one of the significant parameters for calculating aerodynamic forces or instantaneous power required to move wings [20]. In a previous study, the wing’s mass was measured by placing tens of wings using analytical balance [21]; however, it involves a laborious process, such as the prevention of evaporation from the wing’s surface. Our proposed method can easily measure only one single wing weight without such a procedure. The body mass of fruit flies is also useful in the field of biology, such as in the investigation of the relationship between growth temperature and body mass [22], the effect of di(2-ethylhexyl) phthalate (DEHP) on offspring weight changes [23], and genes and genetic pathways related to obesity [24]. Therefore, the proposed method of measuring the mass of body parts of fruit flies is expected to contribute not only to the mechanical field but also to the biological field.

In this study, a force plate was designed to measure the mass of fruit flies. We can easily change the measurement range and plate size by adjusting the width and number of turns of the spiral springs and the thickness of the film. Even when the design is different, the fabrication process ensures ease, and the advantages of toughness and high resolution persist. In principle, it is possible to produce force plate structures with lower spring constants; however, the lower the spring constant, the more sensitive to the airflow and vibration it becomes. To realize a more sensitive force plate system, constructing a sealed chamber is useful to reduce noise due to airflow. Additionally, one force plate was used from the beginning to the end of the experiment; however, the plate was disposable and could be easily replaced when contaminated by bodily fluids. Furthermore, the measurement range can be easily changed by changing the plate according to the target size.

## 5. Conclusions

We proposed a force plate with a spiral spring structure made of polyimide film to measure the mass of a fruit fly. When placing a target object on the plate, the laser displacement meter under the plate detects the vertical displacement of the plate center such that the mass is calculated from the displacement via Hooke’s law. The mechanical properties of polyimide, a material with a low Young’s modulus and viscoelasticity, and a spiral spring structure produced a large displacement. A resolution of less than 10 nm in the laser displacement meter resulted in a mass resolution of less than 2 µg, which was sufficiently sensitive for measuring the wing mass of fruit flies. The fabricated device was remarkably user-friendly because the plate was not damaged when dropped or impacted and was disposable owing to the simple fabrication process. In addition, the parameters of the structure can be easily changed such that the device can be applied to any measurement range and target size. From the measurement of fruit fly mass, we found a strong correlation between body length and body mass and that the proportion of head and thorax/abdomen masses differed between males and females. Therefore, the proposed device proved to be useful for measuring the mass of small insects and other light objects with sub-milligram mass.

## Figures and Tables

**Table 1:** Simulation characteristics of each force plate.

	Spring Constant (N/m)	Resonant Frequency (Hz)
(i)	0.320	16.57
(ii)	0.262	15.26
(iii)	0.221	13.87
(iv)	0.204	13.58

**Table 2:** Experimental characteristics of the fabricated force plate.

	Spring Constant (N/m)	Resonant Frequency (Hz)
(i)	0.329	18.4
(ii)	0.221	15.0
(iii)	0.208	14.3
(iv)	0.205	14.9

**Table 3:** Average measurements of each male and female.

	Body Length (mm)	Wing Length (mm)	Body Mass (mg)	Head (mg)	Thorax/Abdomen (mg)	Wing (mg)
Male	2.26	1.92	0.702	0.0854 (12.55%)	0.527 (77.32%)	0.00279 (0.41%)
Female	2.75	2.13	1.09	0.121 (10.97%)	0.872 (79.50%)	0.00417 (0.39%)

## Data Availability

The data presented in this study are available on request from the corresponding author.
